# Geriatric syndromes, multimorbidity, and disability overlap and increase healthcare use among older Chinese

**DOI:** 10.1186/s12877-018-0840-1

**Published:** 2018-06-25

**Authors:** Johnny T. K. Cheung, Ruby Yu, Zimu Wu, Samuel Y.S. Wong, Jean Woo

**Affiliations:** 1 Institute of Ageing, The Chinese University of Hong Kong, Shatin, Hong Kong; 2 Department of Medicine & Therapeutics, Faculty of Medicine, The Chinese University of Hong Kong, Shatin, Hong Kong; 3 School of Public Health and Primary Care, Faculty of Medicine, The Chinese University of Hong Kong, Shatin, Hong Kong

**Keywords:** Geriatric, Multimorbidity, Disability, Overlap, Outpatient, Hospital admission, Health service use

## Abstract

**Background:**

Geriatric syndromes, multimorbidity, and disability are prevalent among ageing population. However, no study empirically examined their additive or synergistic effect on healthcare use. The present study aims to estimate overlapping prevalence of geriatric syndromes, multimorbidity, and disability; and to examine associations of these three conditions with healthcare use.

**Methods:**

A cross-sectional study was conducted in community-dwelling older adults aged 60 and above in 12 Hong Kong districts. Pearson’s chi-squared test for trend was performed to examine prevalence of geriatric syndromes, multimorbidity, and disability across three age groups (60–69, 70–79, and ≥ 80). Multiple logistic regression was conducted to explore associations of these three conditions with three types of healthcare use (hospital admission, general outpatient clinic and specialist outpatient clinic attendance) respectively.

**Results:**

Among 2618 participants, 75.3, 41.8, and 22.5% had geriatric syndromes, multimorbidity, and disability respectively, and 10.4% had all the three conditions. Prevalence of the three conditions and their coexistence significantly increased with age (*p* for trend < .001). Each condition was independently associated with at least two out of three types of healthcare use. Interestingly, the associations of multimorbidity and disability with specialist outpatient clinic attendance were weakened at older age, while the associations of geriatric syndromes with hospital admission and specialist outpatient clinic attendance were strengthened. Furthermore, the odds of all the three types of healthcare use increased with the number of conditions present (*p* for trend < .001).

**Conclusions:**

Our findings support that the three conditions overlap and increase healthcare use. Early identification, prevention and intervention targeting older adults living with multiple healthcare needs are necessary.

## Background

Geriatric syndromes are a range of conditions representing multiple organ impairment in older adults [1]. Unlike traditional chronic diseases, these syndromes cannot fit into discrete disease categories and are loosely defined [1]. However, frailty [2], sarcopenia [3], cognitive impairment [4], and urinary incontinence [5] are widely recognized as common examples of geriatric syndromes.

Geriatric syndromes, multimorbidity, and disability are closely interrelated. Multimorbidity, the co-occurrence of two or more chronic diseases, can result in geriatric syndromes [6]. Multimorbidity and geriatric syndromes can further cause disability [6]. For example, heart failure and other morbidities accelerate muscle loss leading to sarcopenia, which further result in rapid functional decline [7]. Due to their close interrelation, some researchers considered disability and multimorbidity as geriatric syndromes [8, 9], whereas others suggested that three conditions overlap but are distinct clinical entities [10]. Nevertheless, both definitions highlight the multiple healthcare needs of older adults.

Geriatric syndromes, multimorbidity, and disability are associated with healthcare use. Multimorbidity is traditionally considered as the most important determinant of healthcare use [11], responsible for two-thirds of healthcare cost in America [12]. Hospitalization is strongly associated with severe disability [13]. Geriatric syndromes can predict hospital admission [9, 14]. Furthermore, a review article suggested that these three interrelated conditions may have additive or synergistic effects on healthcare use [10]. In light of the growing healthcare needs of the ageing population, multimorbidity and disability are widely recognized. However, geriatric syndromes are seldom managed in clinical routine [15].

There are knowledge gaps regarding (1) coexistence of geriatric syndromes, multimorbidity and disability within same individuals and (2) their respective associations with healthcare use. First, although several studies estimated overlapping prevalence of frailty, multimorbidity, and disability in community-dwelling older adults [10, 16–20], only one was conducted in Chinese population [21]. In addition, no geriatric syndromes other than frailty were included in these studies. Lack of identification of their multiple healthcare needs may delay local prevention and intervention. Second, although previous studies have explored independent associations of the three conditions with healthcare use [14], none examined whether the associations vary with age. More importantly, the additive or synergistic effect of these three conditions on healthcare use has not been examined empirically. Given the interrelation among the three conditions, the poor understanding of their associations with healthcare use may hinder appropriate long-term care management.

Recognizing the significance of but the dearth of research, we conducted a cross-sectional study on community-dwelling older Chinese in Hong Kong to (1) estimate overlapping prevalence of geriatric syndromes, multimorbidity, and disability; and to (2) examine their associations with healthcare use.

## Methods

### Study background and subject

This cross-sectional study is a part of baseline well-being assessment of the *Jockey Club Community eHealth Care Project*, a telecare programme promoting preventive healthcare and self-management. Specifically, the programme consists of (1) data sharing to a nurse team by cloud technology for proactive monitoring and follow-up, (2) regular health measurement with smart cards for recording, and (3) nursing caring call and regular outreach visits by a multi-disciplinary team including nurses, health workers, and social workers. All community-dwelling older adults aged 60 and above were eligible to participate in this telecare programme. We recruited participants in 24 elderly centres located at 12 districts of Hong Kong, between September 2016 and October 2017.

### Data collection procedure

Each participant was given a tablet to complete an electronic survey, in a group of 6 to 8. At least one staff member of each elderly centre was trained to lead the groups by going through each question with the participants. Other staff members would further assist the participants with survey completion if necessary. Data collected were automatically uploaded to and stored in the cloud.

### Measures

Geriatric syndromes included in this survey were (a) frailty, (b) sarcopenia, (c) mild cognitive impairment, and (d) urinary incontinence. Frailty was measured by the FRAIL scale [22], which consists of five items including fatigue, resistance, ambulation, illness, and loss of weight. The scores ranged from 0 (best) to 5 (worst), representing frail (3–5), pre-frail (1–2), or robust (0) status. The validated SARC-F scale [23] was adopted for sarcopenia screening. The scale consisted of five components, including strength, assistance with walking, rise from a chair, climb stairs, and fall. The scores ranged from 0 to 10, with 0 to 2 points for each component. Scores ≥ 4 indicated the presence of sarcopenia. Mild cognitive impairment was screened with the validated five-item Abbreviated Memory Inventory for Chinese (AMIC) [24]. The scores ranged from 0 to 5, with 1 point for each item. Scores ≥ 3 indicated the presence of mild cognitive impairment. Older adults with any one of the syndromes were considered as living with geriatric syndrome.

Data regarding the presence of chronic diseases diagnosed by doctors was obtained through participants’ self-report. The chronic diseases included (a) hypertension, (b) diabetes mellitus, (c) hypercholesterolemia, (d) heart disease, (e) stroke, (f) chronic obstructive pulmonary disease, and (g) renal disease. Multimorbidity was defined as having two or more chronic diseases.

Disability was determined by the validated Chinese-version five-item Instrumental Activity of Daily Living (IADL) adopted from the Lawton IADL scale [25]. The IADL tasks examined include ability to use telephone, shopping, food preparation, transportation, and ability to handle finance. Participants who had difficulty in performing any one of the activities were classified as living with disability.

Participants reported their healthcare use for any causes in the past 12 months, including (a) hospital admission, (b) general outpatient clinic (GOPC) attendance, and (c) specialist outpatient clinic (SOPC) attendance by responding to a “yes” “no” answer.

Sociodemographic variables including age, gender, marital status, education attainment, and living arrangement were recorded.

### Data analysis

Descriptive statistics including prevalence of geriatric syndromes, multimorbidity, disability, and coexistence of these three conditions were computed. Pearson’s chi-squared test for trend was performed to examine trends in the prevalence rates across three age groups (60–69, 70–79, and ≥ 80). Additionally, strength of interrelations among the three conditions was determined by Cramer’s V. A Cramer’s V of < .1 was considered as weak, .1–.3 as moderate, > .3 as strong.

Multiple logistic regression was conducted to explore associations of the three conditions with hospital admission, GOPC attendance, and SOPC attendance in two approaches. First, geriatric syndromes, multimorbidity, and disability were included in the multivariate analysis for each of the three types of healthcare use. The analyses were then stratified by the three age groups. Second, associations of number and combination of conditions with the healthcare use were explored. Pearson’s chi-squared test for trend was performed to examine trends in healthcare use over increasing number of conditions. All multivariate analyses were further adjusted for the sociodemographic variables. Cases with missing data of any variables (*n* = 3) were excluded from the regression analyses.

Adjusted odds ratios (AORs) and 95% confidence intervals (95% CI) were reported. A *p*-value of < .05 was considered as statistically significant. All statistical analyses were performed by IBM SPSS Statistics 24 and weighted for age and gender.

## Results

Table 1 presents descriptive statistics of 2618 participants. The majority of them were aged 60–69, female, married, had primary education level and lived with others. Mild cognitive impairment was the most common geriatric syndrome (68.3%). GOPC was the most frequently used healthcare service (83.1%).

**Table 1:** Descriptive statistics of participant characteristics (*n* = 2618)

Characteristics	Number	Percent
Age		
60–69	1406	53.7
70–79	672	25.7
≥ 80	540	20.6
Gender		
Male	1245	47.5
Female	1373	52.5
Marital status		
Single	182	7.0
Married	1521	58.1
Widowed	687	26.2
Divorced/separated	227	8.7
Education		
No schooling	443	16.9
Primary	1169	44.7
Secondary	839	32.1
Tertiary	165	6.3
Living arrangement		
Living with others	1867	71.4
Living alone	749	28.6
Chronic disease		
Hypertension	1676	64.0
Diabetes	783	29.9
Hypercholesterolemia	701	26.8
Heart disease	352	13.5
Stroke	143	5.4
Chronic Obstructive Pulmonary Disease	65	2.5
Renal disease	40	1.5
Disability		
IADL	589	22.5
Geriatric syndrome		
Frailty	369	14.1
Sarcopenia	263	10.0
Mild cognitive impairment	1787	68.3
Urinary incontinence	773	29.6
Healthcare use (in the past 12 months)		
Hospital admission	617	23.6
GOPC attendance	2174	83.1
SOPC attendance	2038	77.9

*IADL* Instrumental Activities of Daily Living, *GOPC* general outpatient clinic, *SOPC* specialist outpatient clinic

Figures were weighted by age and gender

### Prevalence of geriatric syndromes, multimorbidity, and disability

**Fig. 1:**
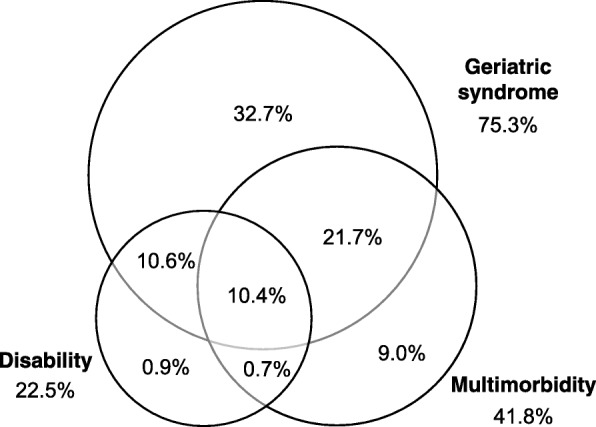
Venn diagram displaying prevalence of geriatric syndromes, multimorbidity, and disability. Cramer’s V: .081 (*p* < .001) for multimorbidity-disability pair; .026 (*p* = .178) for multimorbidity-geriatric syndromes pair; .219 (*p* < .001) for geriatric syndromes-disability pair

Figure 1 is a Venn diagram displaying prevalence of geriatric syndromes, multimorbidity, and disability. The prevalence were 75.3, 41.8, and 22.5% respectively. Overlapping prevalence of the three conditions was 10.4%. The interrelations within the three conditions were weak-to-moderate (Cramer’s V .026–.219).

**Fig. 2:**
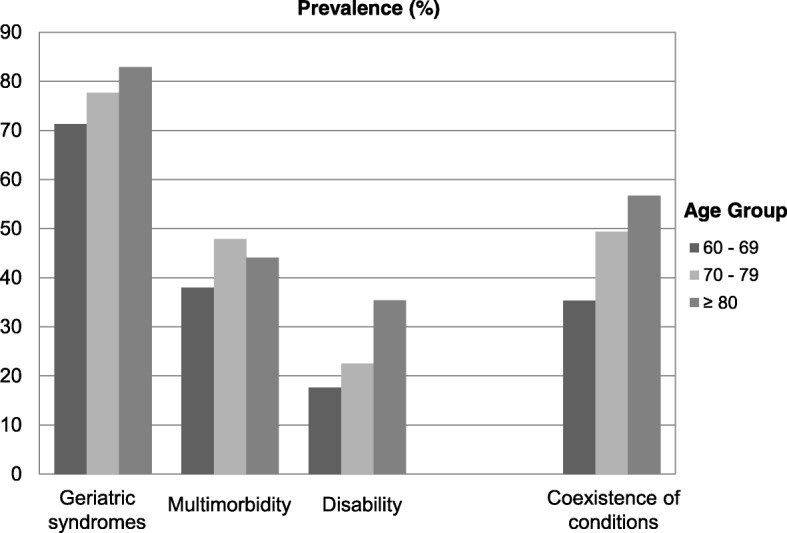
Prevalence of geriatric syndromes, multimorbidity, disability, and coexistence of conditions by age groups. All trends in prevalence across age group were significant at *p* < .001. Coexistence of conditions refers to presence of two or more conditions (geriatric syndromes, multimorbidity, and disability) within same participant

Figure 2 shows prevalence of geriatric syndromes, multimorbidity, disability, and coexistence of conditions by age groups. All the three conditions and their coexistence within same individuals increased in prevalence over age (*p* for trend < .001). Yet, the increasing trend in prevalence of multimorbidity was inconsistent from age group 70–79 to ≥ 80.

### Associations of geriatric syndromes, multimorbidity, and disability with healthcare use

**Fig. 3:**
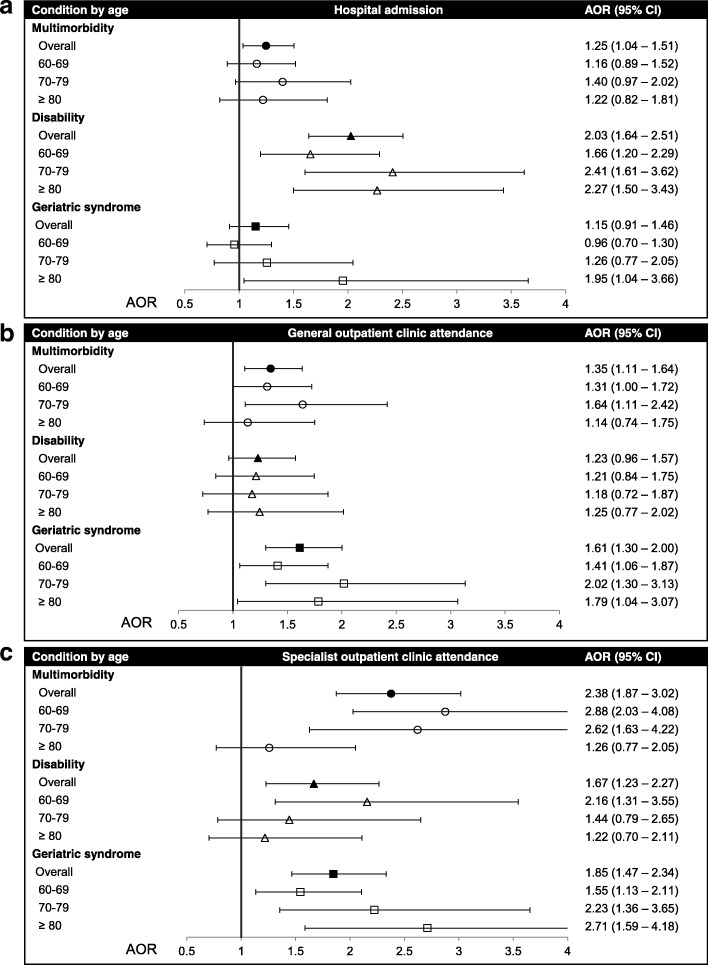
Multiple logistic regression of healthcare use on geriatric syndromes, multimorbidity, and disability. **a** Hospital admission; **b** General Outpatient Clinic (GOPC) attendance; **c** specialist outpatient clinic (SOPC) attendance. Analyses were further adjusted for age, gender, marital status, education, and living arrangement. Reference groups were participants without any conditions

Figure 3 demonstrates results of multiple logistic regression of healthcare use on geriatric syndromes, multimorbidity, and disability. Each condition was independently associated with hospital admission, GOPC attendance and/or SOPC attendance (AOR 1.25–2.38, 95% CI 1.04–3.02). Stratified by the three age groups, the associations of multimorbidity and disability with SOPC attendance were weakened from age group 60–69 to ≥ 80 (AOR decreased from 2.88 to 1.26 for multimorbidity and from 2.16 to 1.22 for disability). At the same time, the associations of geriatric syndromes with hospital admission and SOPC attendance were strengthened in older age groups (AOR increased from 0.96 to 1.95 for hospital admission and from 1.55 to 2.71 for SOPC attendance).

**Fig. 4:**
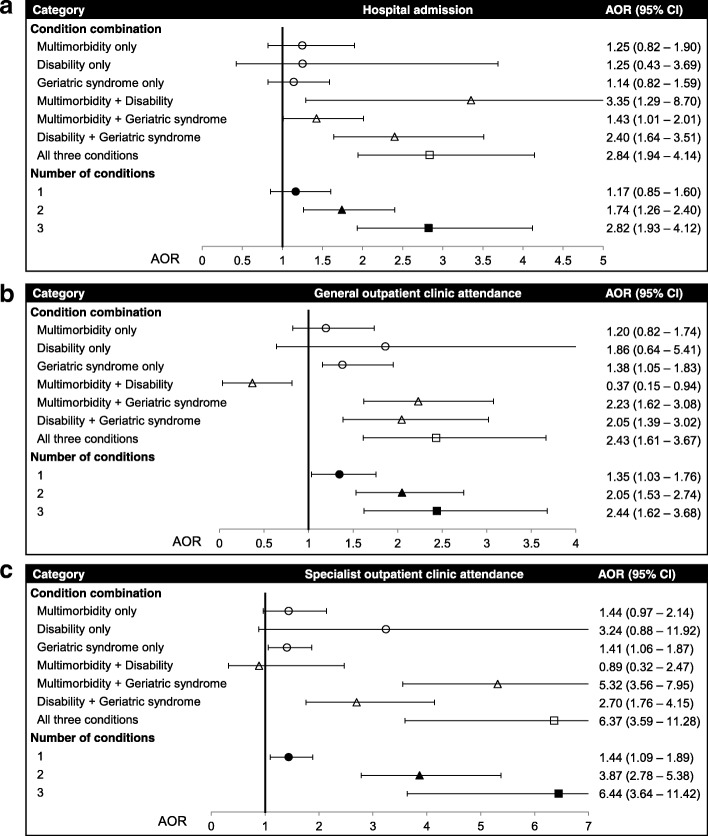
Multiple logistic regression of healthcare use on number and combination of conditions. **a** Hospital admission; **b** General Outpatient Clinic (GOPC) attendance; **c** specialist outpatient clinic (SOPC) attendance. Analyses were further adjusted for age, gender, marital status, education, and living arrangement. Reference groups were participants without any conditions. All trends in healthcare use over increasing number of conditions were significant at *p* < .001

Figure 4 presents results of multiple logistic regression of healthcare use on number and combination of conditions. Dose-response relationships between the number of conditions present and all three types of healthcare use were found (AOR 1.17–1.44, 95% CI 0.85–1.89, for one condition; AOR 1.74–3.87, 95% CI 1.26–5.38 for two conditions; AOR 2.44–6.44, 95% CI 1.62–11.42 for three conditions; all *p* for trend < .001). Specifically, older adults living with geriatric syndromes only (without multimorbidity and disability) had significantly more GOPC and SOPC attendance (AOR 1.38–1.41, 95% CI 1.05–1.87). In contrast, those living with both multimorbidity and disability (without geriatric syndromes) had lower GOPC attendance (AOR 0.37, 95% CI 0.15–0.94).

## Discussion

### Prevalence of geriatric syndromes, multimorbidity, and disability

In our sample, 75.3, 41.8, and 22.5% had the three conditions respectively, and 10.4% had all the three conditions (Fig. 1). Using similar assessment tools and classifications of the three conditions, a study reveals similar prevalence rates of frailty (10.6%), multimorbidity (46.3%), and disability (25.0%) among Iceland older adults [16]. Meanwhile, the high prevalence of geriatric syndromes found in this study was accounted for by that of mild cognitive impairment (68.3%) diagnosed, much higher than that in other Asian countries such as Japan [26] and Korea [27]. The high prevalence of mild cognitive impairment revealed in our study might be attributed to the relatively low specificity of AMIC (57.4%).

It is notable that geriatric syndromes, multimorbidity, disability, and their coexistence increased in prevalence over age (Fig. 2). On the one hand, the increasing prevalence of geriatric syndromes [28], multimorbidity [29], and disablity [30] over age were well-documented. On the other hand, what our study adds is that ageing strengthened the coexistence of the three conditions in community-dwelling older adults.

Furthermore, our study may also provide some insights regarding the definition of geriatric syndromes. The weak-to-moderate associations among geriatric syndromes, multimorbidity, and disability (Cramer’s V: .026–.219) supported that the three conditions are different clinical entities but cannot be totally untangled.

### Associations of geriatric syndromes, multimorbidity, and disability with healthcare use

This is the first study to explore whether associations of the three conditions with healthcare use vary with age and with coexistence of conditions. As expected, this study demonstrates independent associations of the three conditions with higher healthcare use (Fig. 3), in line with previous findings [14]. But interestingly, in our age-stratified analysis, the associations of multimorbidity and disability with SOPC attendance were weakened in older age groups (Fig. 3c). Conversely, the associations of geriatric syndromes with hospital admission and SOPC attendance were strengthened at advanced age (Fig. 3a and c). There were two possible underlying reasons. First, as there was an inconsistent increase in multimorbidity over age observed (Fig. 1), survival bias might play a role in the age-related prevalence of the multimorbidity. Alternatively, instead of attending outpatient clinics, older adults with multimorbidity and/or disability might live in elderly homes to receive care. In Hong Kong, eligible criteria for elderly home admission include advanced age (65 and above), requiring medical care, and disability [31]. Meanwhile, geriatric syndromes are not specified as the admission criteria. Therefore, older adults aged 70 and above with multimorbidity and disability requiring medical care might be underrepresented in our community sample.

Our study also showed dose-response relationships between the number of conditions present and all three types of healthcare use were observed (Fig. 4). The results empirically support additive or synergistic effects of the conditions on healthcare use. Additionally, older adults living with geriatric syndromes only (without multimorbidity and disability) have significantly higher GOPC and SOPC attendance. By contrast, contradicting the notion, those living with multimorbidity and disability (without geriatric syndromes) had lower GOPC attendance. In light of a strong association of the multimorbidity-disability pair with hospital admission (Fig. 4a), older adults living with both multimorbidity and disability might have medical conditions severe enough for direct hospital admission, instead of attending outpatient clinics.

### Limitations

Our study has several limitations. First, the findings drawn from community-dwelling older adults might not be generalizable to those living in elderly homes. Second, chronic diseases and geriatric syndromes assessed in this study were limited. The prevalence of the conditions might be underestimated. Third, our results relied on self-reported data, which were subject to recall bias and cognitive impairment in older adults. Fourth, the cross-sectional study design cannot confirm causations but only associations between the conditions and the healthcare use. Fifth, confounders including lifestyle and long-term medications were not adjusted in the multivariate analysis.

## Conclusions

Our study reveals that geriatric syndromes, multimorbidity, and disability overlapped and increasingly overlapped at advanced age. The three conditions were independently and cumulatively associated with higher inpatient and outpatient use. These findings inform policy making for early identification of multiple healthcare needs of older adults, in order to facilitate early prevention and intervention. Future studies should adopt longitudinal study design and include older adults living in elderly homes. Mechanisms underlying interrelation or hierarchical relation among the three conditions can also be explored. Health service research can examine community screening and clinical management models targeting the older adults living with multiple conditions including geriatric syndromes.

## Abbreviations

95% CI: 95% confidence interval
AMIC: Abbreviated Memory Inventory for Chinese
AOR: Adjusted odds ratio
GOPC: General outpatient clinic
IADL: Instrumental activity of daily living
SOPC: Specialist outpatient clinic
